# Domesticating *Vigna stipulacea*: Chromosome-Level genome assembly reveals *VsPSAT1* as a candidate gene decreasing hard-seededness

**DOI:** 10.3389/fpls.2023.1119625

**Published:** 2023-04-17

**Authors:** Yu Takahashi, Hiroaki Sakai, Hirotaka Ariga, Shota Teramoto, Takashi L. Shimada, Heesoo Eun, Chiaki Muto, Ken Naito, Norihiko Tomooka

**Affiliations:** ^1^ Research Center of Genetic Resources, National Agriculture and Food Research Organization, Tsukuba, Japan; ^2^ Research Center of Advanced Analysis, National Agriculture and Food Research Organization, Tsukuba, Japan; ^3^ Institute of Crop Science, National Agriculture and Food Research Organization, Tsukuba, Japan; ^4^ Graduate School of Horticulture, Chiba University, Matsudo, Japan; ^5^ Plant Molecular Science Center, Chiba University, Inage-ku, Japan

**Keywords:** *de novo* domestication, genus *Vigna*, legume, physical seed dormancy, wild species, hard-seededness

## Abstract

To increase food production under the challenges presented by global climate change, the concept of *de novo* domestication—utilizing stress-tolerant wild species as new crops—has recently gained considerable attention. We had previously identified mutants with desired domestication traits in a mutagenized population of the legume *Vigna stipulacea* Kuntze (minni payaru) as a pilot for *de novo* domestication. Given that there are multiple stress-tolerant wild legume species, it is important to establish efficient domestication processes using reverse genetics and identify the genes responsible for domestication traits. In this study, we identified *VsPSAT1* as the candidate gene responsible for decreased hard-seededness, using a *Vigna stipulacea isi2* mutant that takes up water from the lens groove. Scanning electron microscopy and computed tomography revealed that the *isi2* mutant has lesser honeycomb-like wax sealing the lens groove than the wild-type, and takes up water from the lens groove. We also identified the pleiotropic effects of the *isi2* mutant: accelerating leaf senescence, increasing seed size, and decreasing numbers of seeds per pod. While doing so, we produced a *V. stipulacea* whole-genome assembly of 441 Mbp in 11 chromosomes and 30,963 annotated protein-coding sequences. This study highlights the importance of wild legumes, especially those of the genus *Vigna* with pre-existing tolerance to biotic and abiotic stresses, for global food security during climate change.

## 1 Introduction

Owing to the effects of climate change on food production, *de novo* domestication of stress-tolerant wild species has recently gained considerable attention (Fernie and Yan, 2019; Gasparini et al., 2021; Razzaq et al., 2021). Feasibility studies have been conducted in which domestication-related traits using new genetic technologies, such as genome editing or reverse genetic approach to mutagenesis, have been introduced in wild grass and vegetable species (Shapter et al., 2013; Dorn et al., 2015; Zsögön et al., 2018; Yu et al., 2021). In legumes, wild species of the genus *Vigna* Savi are candidates for *de novo* domestication, as they demonstrate remarkable tolerance to biotic and abiotic stresses, including salinity, drought, high/low pH, and flooding (Tomooka et al., 2014). We previously reported the first step of domesticating *Vigna stipulacea* Kuntze by ethyl methanesulfonate mutagenesis followed by phenotype screening (Takahashi et al., 2019). This species inhabits mainly South Asia and has a short growing period, heat tolerance, and resistance to multiple biotic stresses (Tomooka et al., 2011; van Zonneveld et al., 2020). Its seeds are edible, and it is mainly locally cultivated as pasture but sometimes as food. However, only a few farmers cultivate *V. stipulacea* because of the high labor caused by the strong behavior of pod-shattering and hard-seededness, which needs to be released for uniform germination (Tomooka et al., 2011). To improve these traits, we screened and obtained mutants with reduced pod-shattering and with decreased hard-seededness (Takahashi et al., 2019). As there are multiple stress-tolerant wild legumes, it is important to establish a domestication process with new genetic technologies. However, this process requires the identification of genes relevant to desired domestication-related traits.

One such trait is seed dormancy, defined by Foley (2001) as “the temporary failure of a viable seed to germinate, after a specific length of time and in a particular set of environmental conditions that allow germination after the restrictive state has been terminated by either natural or artificial conditions (Simpson, 1990).” It is an adaptive trait for the temporal control of germination in response to variable external environments. There are several types of seed dormancy including physical, mechanical, and chemical inhibition by the outer layers of the embryo, inability to germinate because of an undifferentiated or immature embryo, and repression of germination by metabolic restraints (Bewley et al., 2013). Among these, physical seed dormancy, identified in 18 angiosperms, is characterized by water impermeability of seed or fruit coats (Gama-Arachchige et al., 2013). Hard-seededness, the primary cause of physical seed dormancy in legumes, is provided by a palisade layer of lignified macrosclerids, impregnated with water-repellent phenolic and suberin-like compounds. Hard-seededness is overcome by opening water gap structures, such as the lens in legumes (Steinbrecher and Leubner-Metzger, 2018). Moreover, temperature- and moisture-dependent stimuli cause irreversible opening of the water gap.

Major domesticated legumes have decreased hard-seededness which has been associated with one to six loci, depending on species, during domestication (Smýkal et al., 2014). One of the genes controlling hard-seededness in soybeans (*Glycine max* (L.) Merr.) is *GmHs1-1*, which encodes a calcineurin-like transmembrane metallophosphoesterase associated with seed coat calcium content (Sun et al., 2015). In the common bean (*Phaseolus vulgaris* L.), a 5-bp frameshift in the gene encoding pectin acetylesterase-8-2 (*PAE8-2*) has been reported as a candidate causal variant that accelerates lens-specific water uptake by expanding microcracks within the lens groove (Soltani et al., 2021). However, it is unclear whether such single mutations are sufficient to decrease hard-seededness in wild species. In barrel clover (*Medicago truncatula* Gaertn.), a loss of function mutation in a class II KNOTTED-like homeobox (*KNOX*) gene, *KNOX4*, forms dysfunctional palisade cuticular layer due to altered composition of lipid monomer in the seed coat, leading to decreased hard-seededness (Chai et al., 2016).

We have previously identified three *V. stipulacea* mutants that decrease hard-seededness—*increased seed imbibition 1* (*isi1*), *isi2*, and *isi3*—from ethyl methanesulfonate mutagenesis (Takahashi et al., 2019). The *isi1* mutant begins taking up water through the entire seed coat within minutes of watering whereas the *isi2* mutant takes up water through the seed’s lens, requiring a week before 80% of seeds complete water uptake. We also identified *VsCESA7*, which encodes a putative ortholog of cellulose synthase A catalytic subunit 7 in *Arabidopsis* (*AtCESA7*/*IRX3*/*MUR10*, AT5G17420), as a candidate gene for the *isi1* phenotype (Takahashi et al., 2019) using whole-genome sequencing, which facilitated bulked segregant analysis. However, it was challenging to perform bulked segregant analysis in our previous study because the *V. stipulacea* genome was fragmented, with 2,102 scaffolds. In such cases, it is important to obtain chromosome-level assemblies.

In the present study, we report another bulked segregant analysis that allowed us to identify a gene responsible for the *isi2* phenotype. We constructed a chromosome-level genome assembly, with annotation of 30,963 protein-coding genes. We then resequenced the bulked segregant derived from wild-type and the *isi2* individuals, to identify *VsPSAT1*, a putative ortholog of *Arabidopsis PSAT1*, encoding phospholipid sterol acyltransferase 1.

## 2 Materials and methods

### 2.1 Plants

We used *V. stipulacea* (accession JP252948 in NARO Genebank) and its ethyl methanesulfonate-induced *isi2* mutant (Takahashi et al., 2019). Plants were cultivated in 5-L pots with seven biological replicates in a single greenhouse maintained at 25°C or higher. Plants were scored for days to flowering, height 46 days after sowing, largest terminal leaflet width and length, shoot dry weight, seeds per pod, pod length, 100-seed weight, and water uptake in 20 seeds per individual following storage at 4°C for 1, 6, and 24 months after harvest. We counted the number of seeds taking up water at 1, 2, 3, 7, 10, 14, 21, and 28 days after watering. Data for one-month-old seeds and six-month-old seeds were published in our previous study (Takahashi et al., 2019).

We used the SPAD chlorophyll meter (SPAD-502, KONICA MINOLTA, INC., Tokyo, Japan) to measure early senescence. Fully expanded third terminal leaflets were detached from plants cultivated for one month in 5-L pots with 5 biological replicates in a single greenhouse maintained at 30°C or lower, then incubated on the water surface in 9-cm Petri dishes for 28 days under a cycle of 12 h of light at 25°C and 12 h of darkness at 22°C.

### 2.2 X-Ray computed tomography

Water entry points were identified using the method of Soltani et al. (2021). Seeds were submerged in Lugol solution containing 33 g/L of I_2_ (092-05422, FUJIFILM Wako Pure Chemical, Tokyo, Japan) and 66 g/L of KI (160-03952, FUJIFILM Wako Pure Chemical) as a contrasting agent for 6 h at 25°C, followed by rinsing with water and drying with a paper towel. Seeds were imaged using an inspeXio SMX-225CT FPD HR (Shimadzu Corporation, Kyoto, Japan). Each scan included 1200 projections, averaging two frames over 360° (pixel detector resolution 3000 × 3000), at 4 frames per second. Finally, 1024 × 1024-pixel slices were computed at a final spatial resolution of 15 μm. Tube voltages of 75 kV and tube currents of 100 μA were used. Metal filters were not used during imaging. Reconstructed CT volumes were visualized using VG Studio MAX 3.2 software (Volume Graphics, Heidelberg, Germany). Regions with strong signal intensity were assumed to be Lugol solution-permeated.

### 2.3 Scanning electron microscopy

Seed surface morphologies were analyzed using a JEOL JSM-5610 instrument (LV, Tokyo, Japan). Samples were coated using a fully automated sputter coater (JFC-1600, JEOL Ltd., Tokyo, Japan) with fine-grained platinum for 100 s at 80 mA. The hilum side and the lens groove were photographed at 50X and 500X magnifications, respectively.

### 2.4 Optical microscopy

Before drying, mature seeds were sectioned at 200 μm using a Plant Microtome MTH-1 (Nippon Medical & Chemical Instruments Co., Ltd., Osaka, Japan). Sections were stained with 0.01% toluidine blue O and observed under an ECLIPSE Ci (OLYMPUS, Tokyo, Japan).

### 2.5 Whole-genome sequencing and annotation

Genomic DNA from wild-type plants was extracted using the NucleoBond HMW DNA extraction kit (Macherey-Nagel, Düren, Germany) and sequenced using the PacBio Sequel II platform (Pacific Biosciences, Menlo Park, CA, USA). HiFi reads were created using the ccs and lima tools (https://github.com/PacificBiosciences/pbbioconda) with “–min-passes 5 –min-rq 0.995” and “–min-score 26 –min-length 10000” options, respectively. *De novo* assembly of the HiFi reads was performed using Canu ver. 2.0 (Koren et al., 2017), followed by the removal of haplotigs using Purge_Dups (Guan et al., 2020) with default settings.

To construct a chromosome-level assembly of the wild-type genome, we performed RAD-seq using 384 F2 plants derived from a cross between wild-type and another accession JP252958. Genomic DNA was extracted from young leaves using the CTAB method, digested with *Bgl*II and *Eco*RI, ligated with Y-shaped adaptors, amplified using polymerase chain reaction (PCR) with KAPA HiFi HS ReadyMix (Kapa Biosystems Inc., Wilmington, MA, USA), and size-selected using E-Gel size select (Life Technologies, Carlsbad, CA, USA). Approximately 450-bp library fragments were retrieved. Further details of the library preparation have been previously described (Sakaguchi et al., 2015). Sequencing was performed using the paired-end 151-bp mode of HiSeq X (Illumina Inc., San Diego, CA, USA). Quality filtering was performed using Trimmomatic ver. 0.39 (Bolger et al., 2014). Filtered sequence reads were mapped to contigs using BWA-MEM ver. 0.7.17 (Li and Durbin, 2009) with the “-q 20 -F 0×100” option. Single nucleotide polymorphisms (SNPs) were called using Stacks ver. 2.53 (Rochette et al., 2019) using default settings. Missing genotypes were imputed using ABHGenotypeR (Furuta et al., 2017). We removed SNPs for which genotypes were missing in more than 30% of the 384 samples or allele frequencies deviated from Hardy–Weinberg equilibria (*p* < 1.0E-10). The genetic map was constructed using OneMap software (Margarido et al., 2007) with the “max.rf=0.14” option (for linkage grouping) and the Kosambi map function. SNPs were ordered using three algorithms: “rec,” “rcd,” and “ug.” Chromosome sequences were reconstructed by aligning contigs on the genetic map according to the order of the SNPs.

Protein-coding genes were identified as previously described (Takahashi et al., 2019). For *ab initio*- and transcriptome-based gene prediction, we used the RNA-Seq data published by Takahashi et al. (2019). We combined protein sequence data deposited in UniProt (UniProt Consortium, 2021) with those of 10 *Vigna* species downloaded from the *Vigna* genome server (Sakai et al., 2016): *V. angularis*; *Vigna exilis* Tateishi and Maxted; *Vigna indica* T. M. Dixit, K. V. Bhat, and S. R. Yadav; *Vigna marina* (Burm. f.) Merr.; *Vigna minima* (Roxb.) Ohwi and Ohashi; *Vigna mungo* (L.) Hepper; *Vigna riukiuensis* (Ohwi) Ohwi and Ohashi; *Vigna trilobata* (L.) Verdc.; *Vigna vexillata* (L.) A. Rich.; and *Vigna* sp. (NI1135 in Meise Botanic Garden, Belgium). These data were used to create a non-redundant set of sequences using MMseqs2 (Steinegger and Söding, 2017) with the “–min-seq-id 0.99 -c 0.99 –cov-mode 0” options. We annotated transposable elements using the Extensive *de novo* Transposable Element Annotator with default settings (Ou et al., 2019).

### 2.6 Detecting presence variations

*V. stipulacea* and *V. angularis* genomes were aligned one-to-one using LAST (Frith and Noé, 2014). Furthermore, unaligned regions (≥100 bp) in one species where flanking sequences (≥1000 bp for each end) were aligned with another species with no large gaps (>100 bp) (PVs candidates) were extracted. PV coordinates were determined by aligning each PV and flanking sequences of the two species using MAFFT (Katoh and Standley, 2013).

### 2.7 Genetic analysis

F2 plants were produced by crossing the wild-type with the *isi2* mutant. In total, 288 F2 plants were cultivated in 1-L pots; DNA was extracted from their young leaves, and water uptake in 20 one-month-old seeds 10 days after watering and 20-seed weight (to estimate 100-seed weight) were measured. Five plants exhibiting the wild-type phenotype that did not produce sufficient seeds were excluded from the evaluation of seed weight. A total of 163 F3 plants derived from 11 F2 plants with recombination among the SNP positions 31,842,848, 32,352,997, and 32,970,540 nt on chromosome 4 were cultivated in 0.2-L pots; DNA was extracted from their young leaves, and water uptake in 10 2-week-old seeds 28 days after watering, 10-seed weights (to calculate 100-seed weights), and numbers of seeds per pod for three pods were measured.

### 2.8 Bulked segregant analysis

We adopted the MutMap strategy (Abe et al., 2012) to identify candidate SNPs for the *isi2* phenotype. Two DNA pools of 215 wild-type phenotypes and 73 *isi2* phenotypes in the F2 plants from the wild-type and the *isi2* mutant were sequenced using the paired-end 151-bp mode of HiSeq X (Illumina). Sequence reads were quality-filtered using Trimmomatic and mapped to the genome assembly using BWA-MEM. SNPs were identified using GenotypeGVCFs of GATK ver. 4.2.1.0 (Van der Auwera and O’Connor, 2020) and filtered with the “QD < 2.0 || FS > 60.0 || MQ < 40.0 || MQRankSum < -12.5 || ReadPosRankSum < -8.0” options of VariantFiltration implemented in GATK. The retained variants were used to calculate SNP indices. Each variant was functionally annotated with the SnpEff ver. 4.3t (Cingolani et al., 2012) using annotated gene structures.

### 2.9 Amplicon sequencing

The genotypes for the SNPs at 31,842,848 nt, 32,352,997 nt, and 32,970,540 nt on chromosome 4 were determined for 288 F2 and 163 F3 plants. Primers used for PCR and Sanger sequencing are shown in **Table 1**. For Sanger sequencing, we amplified template DNA using AmpliTaq Gold 360 Master Mix (Thermo Fisher Scientific, Waltham, MA, USA) followed by sequencing with BigDye Terminator v3.1 (Thermo Fisher Scientific) and an ABI Genetic Analyzer 3130xl (Thermo Fisher Scientific) according to the manufacturer’s protocols.

**Table 1:** PCR and sequencing primers.

Target	PCR and *sequencing*		Sequencing
	Left	Right	
SNP at 31,842,848 nt	cctagctgcagcaaatcagagaact	atgggagatatgcaggaaggtaagc	acatgtttgtggatccatgc
SNP at 32,352,997 nt	*ctgaggcgctcttgttatgttcact*	*tgtatgtggaatgaattggggagag*	
SNP at 32,970,540 nt	atcgtttgaattttgtggaaggaca	gaaacagcggttaaagcttttagac	aacatgtggcctgacaatga
RT-PCR for **Figure 4B**	tcttctgcatttatgggtctga	cacttttggtccaagccagt	
RT-PCR for **Supplementary Figure 4**	tcccaaaactttccaactgc	*tcattctcaaggttaacccact*	ctgggtggccaacaaaaata

### 2.10 Reverse transcription (RT)-PCR

Young pods of approximately 5 mm in length 3 days after flowering and unexpanded third leaves were harvested. Total RNA was extracted using the RNeasy Plant Mini Kit (QIAGEN, Hilden, Germany). Vigst.04G198600 cDNA was amplified using the PrimeScript One Step RT-PCR Kit Ver. 2 (TAKARA Bio Inc., Shiga, Japan) with the primers shown in **Table 1**. DNA extracted from unexpanded third leaves was used as a negative control. Electrophoresis was performed using 2% agarose gels.

### 2.11 Orthology analysis

To identify Vigst.04G198600 orthologs in the genomes of closely related legume species, we obtained protein sequences from 11 *Vigna* species from the *Vigna* genome server as described above, and Phytozome (for *V. unguiculata*), as well as those of *P. vulgaris*, *G. max*, and *Glycine soja* Siebold and Zucc. from Phytozome (Goodstein et al., 2012). We ran Orthofinder ver. 2.5.4 (Emms and Kelly, 2019) using default settings to identify the single orthogroup including Vigst.04G198600. We measured collinearity between *V. stipulacea* and each of the legume species using MCScanX and confirmed that all genes in the orthogroup were syntenic with Vigst.04G198600. To construct a phylogenetic tree of orthologs, we created codon alignments and generated a neighbor-joining tree using the Nei–Gojobori method in MEGA ver. 10 (Kumar et al., 2018). Amino-acid sequences were aligned using the Clustal Omega multiple sequence alignment tool (http://www.ebi.ac.uk/Tools/msa/clustalo/).

## 3 Results

### 3.1 Decreased hard-seededness of *isi2*

**Figure 1:**
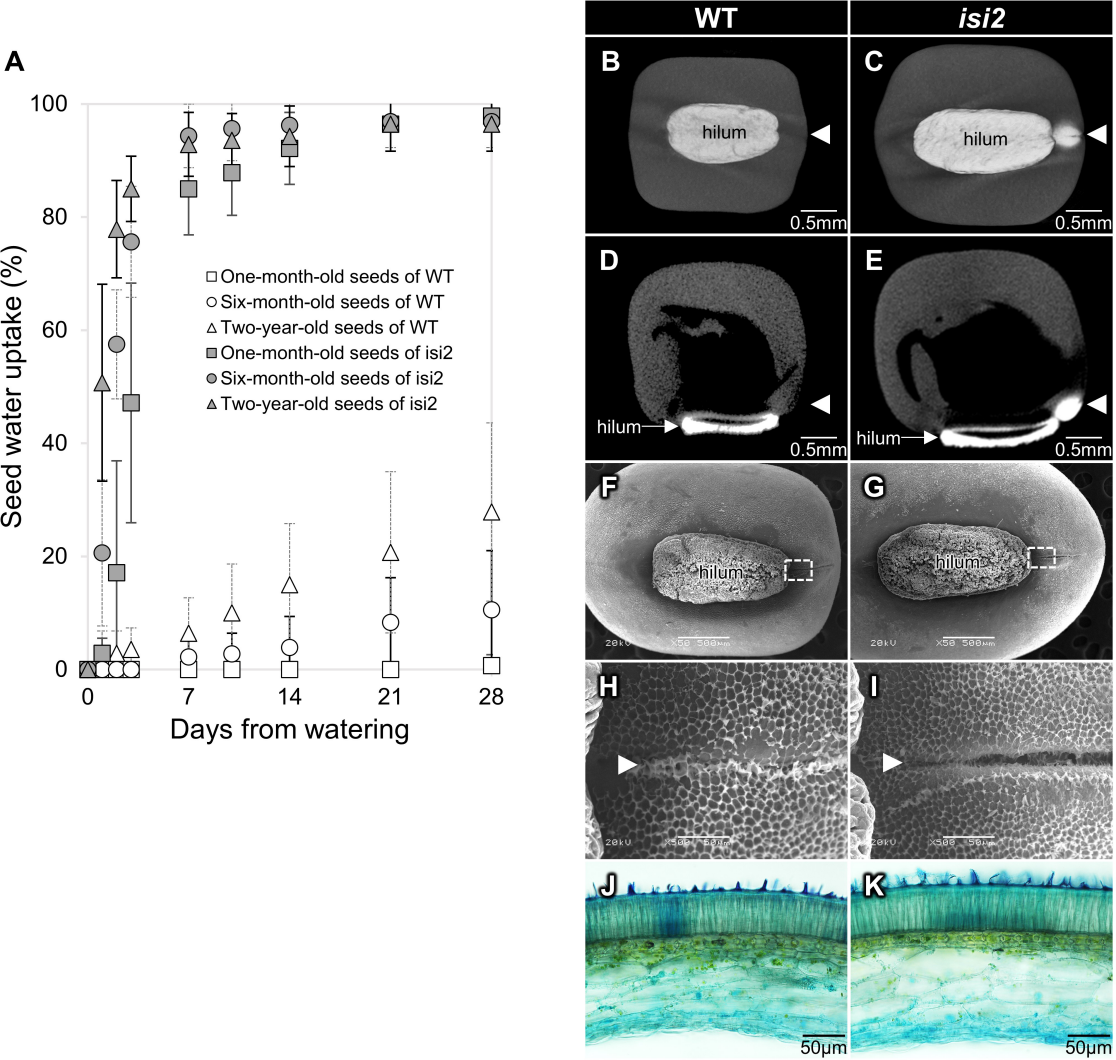
Characterization of the *isi2* mutant. Seed water uptake over time as a function of storage period **(A)**. White shapes indicate the wild-type and gray shapes indicate the *isi2* mutant. Squares, one-month-old seeds; circles, six-month-old seeds; triangles, two-year-old seeds. N=7. 3D-rendered images facing the hilum **(B, C)** and longitudinal sections of centers **(D, E)** from computed tomography (CT) scans of wild-type **(B, D)** and the *isi2* mutant **(C, E)** seeds six hours after submergence in a contrasting agent. White areas indicate the presence of the agent. The white arrowheads indicate the lens. 50X images facing the hilum with a 500 μm scale bar **(F, G)**, and 500X images of the dotted square photographs **F** and **G** with a 50 μm scale bar **(H, I)** from scanning electron microscopy (SEM) of the surfaces of the wild-type **(F, H)** and *isi2* mutant **(G, I)** seeds. White arrowheads indicate the lens groove. Cross sections of coats observed under an optical microscope of wild-type **(J)** and the *isi2* mutant **(K)** seeds.

Seeds preserved at 4°C for one, six, and twenty-four months after harvest were submerged in water. In wild-type *V. stipulacea*, water uptake at one month after watering was 1% in 1-month-old seeds, 11% in 6-month-old seeds, and 28% in 2-year-old seeds. These results indicate that water uptake was low in young seeds but higher in older wild-type seeds (**Figure 1A**, **Supplementary Table 1**). In contrast, water uptake in *isi2* mutant seeds was consistently higher at 96–98% a month after submersion regardless of the storage period but tended to be higher for older seeds until 3 days of submersion (**Figure 1A**, **Supplementary Table 1**).

In addition, CT scanning identified water entry at the lens in the *isi2* mutant (**Figure 1B, C**). Longitudinal sections showed entry into the cotyledon through the lens (**Figure 1D, E**). SEM revealed that the seed coat microstructure of *isi2* was different from that of the wild-type. The mutant had decreased honeycomb-like epicuticular wax deposition in the lens groove compared to the wild-type; however, no differences were observed in the entire seed coat (**Figure 1F-I**, **Supplementary Figure 1**). Similarly, optical microscopy revealed no reproducible differences in seed coat cross sections between the mutant and the wild-type (**Figure 1J, K**). Three biological replicates are shown in the **Supplementary Figure 2**.

### 3.2 Whole-genome sequencing and annotation

We sequenced the wild-type genome and obtained 20.62 Gbp of HiFi reads. *De novo* assembly generated 823 contigs with a total length of 413.48 Mbp and an N50 length of 11.87 Mbp (**Table 2**). We constructed a genetic map of the 11 chromosomes based on 1,163 SNP markers derived from RAD-seq and anchored 44 contigs to it. These anchored contigs spanned 387.57 Mbp, covering 88% of the estimated genome size (441.54 Mbp). The repetitive fraction of the genome was 38% (156.62 Mbp), smaller than that of the *V. angularis* genome (45%). The most abundant class of repetitive elements was *Gypsy* type LTR-retrotransposons (7.5%), followed by *unknown* (6.8%) and *Copia* type (6.4%) LTR-retrotransposons.

**Table 2:** *Vigna stipulacea* genome assembly statistics.

	Value
Contigs	
Number of contigs	823
Total contig length (bp)	413,481,714
N50 contig length (bp)	11,869,769
Mean contig length (bp)	502,408
Chromosome-scale assembly	
Number of anchored contigs	44
Number of anchored base pairs (with gaps) (bp)	387,565,718
Number of unanchored contigs	779
Number of unanchored base pairs (bp)	25,888,147
Genome annotation	
Number of protein-coding genes	30,963
Mean protein length (aa)	421
Complete BUSCOs	1,592 (98.7%)
Complete and single-copy BUSCOs	1,557 (96.5%)
Complete and duplicated BUSCOs	35 (2.2%)
Fragmented BUSCOs	12 (0.7%)
Missing BUSCOs	10 (0.6%)

We identified 30,963 protein-coding genes by combining *ab initio*-based, transcriptome-based, and protein-based approaches (**Table 2**). BUSCO analysis of the embryophyta_odb10 dataset showed 98.7% completeness in the predicted proteins, slightly higher than our previous version (95.8%; Takahashi et al., 2019).

**Figure 2:**
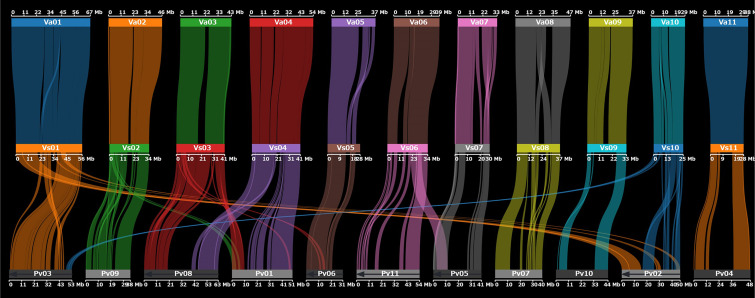
Dot plot showing chromosomal synteny between *V. stipulacea* (middle) and *V. angularis* (top) and between *V. stipulacea* and *P. vulgaris* (bottom).

In addition, we assessed synteny between the genomes of *V. stipulacea* and azuki bean (*Vigna angularis* (Willd.) Ohwi et H. Ohashi) and between *V. stipulacea* and *P. vulgaris*. The genomes of *V. stipulacea* and *V. angularis* were highly syntenic across all 11 chromosomes. We detected 669 synteny blocks between *V. stipulacea* and *V. angularis* with 23,574 (76%) of the 30,963 genes overlapping. In contrast, the genomes of *V. stipulacea* and *P. vulgaris* had several structural differences, including translocations and inversions (**Figure 2**).

Despite the high synteny between *V. stipulacea* and *V. angularis*, precise comparisons based on the whole-genome alignment revealed several structural variations. We detected 6,298 presence variations (PVs) in the *V. stipulacea* genome, with a total length of 5,094,208 bp, whereas the *V. angularis* genome harbored 8,090 PVs with a total length of 9,822,523 bp. Of the 5,094,208 bp of PVs in *V. stipulacea*, 3,154,013 (62%) were annotated as repetitive elements whereas 27 protein-coding genes were annotated in the PV regions.

### 3.3 Mapping of the *isi2* candidate loci

Upon testing the genetic factor, we concluded that the *isi2* phenotype was caused by a mutation in a single locus according to the law of independence. We defined the *isi2* phenotype as one in which more than 80% of seeds exhibited water uptake within one to four weeks after watering. Among F2 (WT × *isi2*) plants, 215 showed the wild-type phenotype and 73 showed the *isi2* phenotype, consistent with the expected 3:1 Mendelian ratio (216:72; *p* = 0.89, based on the Chi-square test; **Supplementary Table 2**).

**Figure 3:**
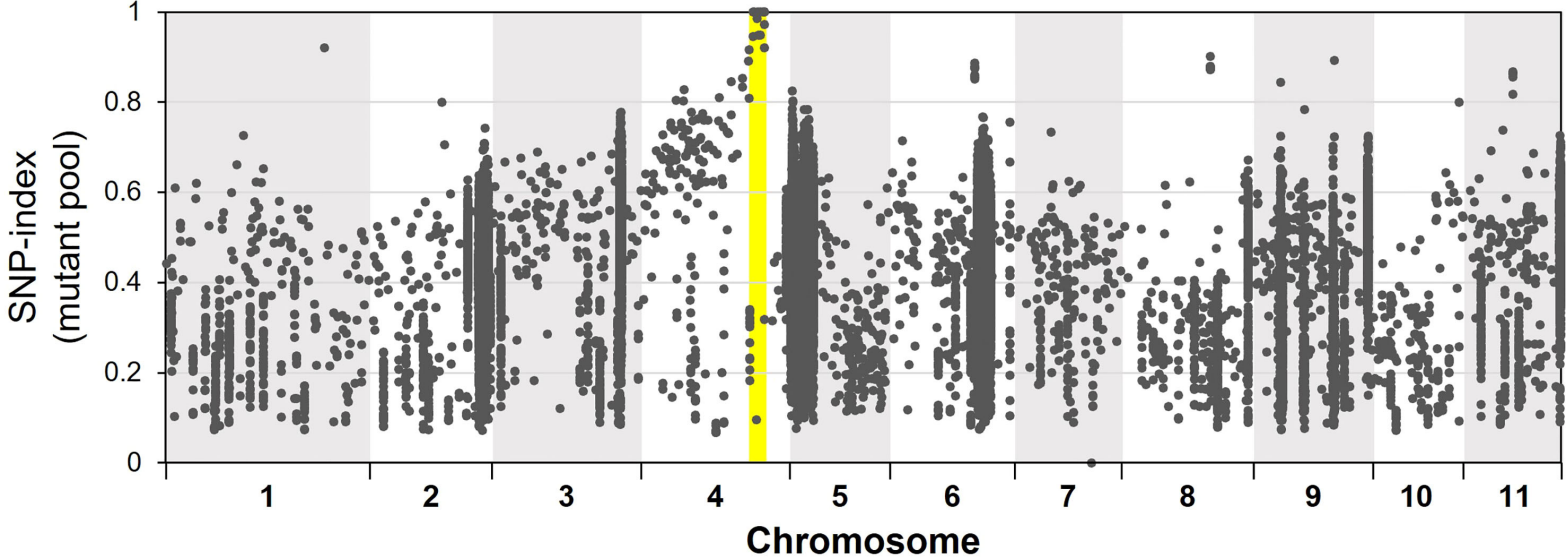
Frequencies of mutant alleles (SNP index) in the mutant pool of the F2 (WT × *isi2*) plants. The x-axis indicates physical positions across chromosomes and the y-axis indicates mutant allele frequencies for each SNP. Yellow highlighting indicates the locus fixed with mutant alleles.

**Figure 4:**
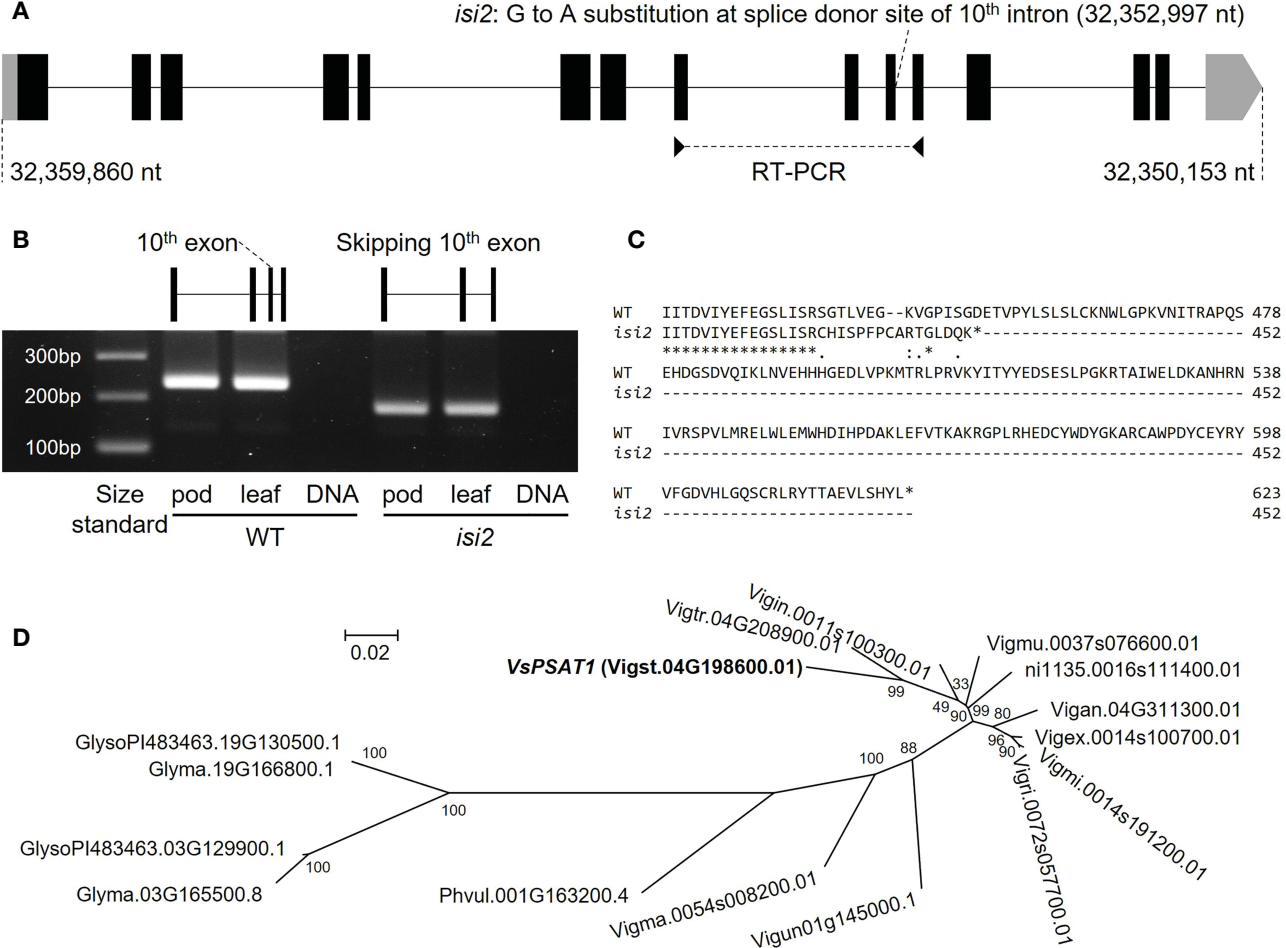
*VsPSAT1* transcripts and putative orthologs. **(A)** Schematic showing differences between wild-type and *isi2* alleles. Boxes indicate exons; lines indicate introns. Coding DNA sequences (CDS) are filled with black. A G>A substitution at the splice donor site between exon 10 and intron 10 is present at 32,352,997 nt. **(B)** RT-PCR products. Primer pairs were designed from the sequences of exons 8 and 11. The schematic shows the skipping of exon 10 in the *isi2* mutant. **(C)** Predicted amino-acid sequences of wild-type and mutant *VsPSAT1*. Asterisks indicate conserved residues. Colons indicate similar residues; periods indicate weakly similar residues. Hyphens indicate gaps introduced to optimize the alignment. **(D)** Phylogeny of putative *PSAT1* orthologs in the tribe Phaseoleae. The operational taxonomic units are amino-acid sequences.

For bulked segregant analysis, we pooled F2 (WT × *isi2*) plants into wild-type and *isi2* phenotypes and sequenced each of them. We identified 61,664 SNPs across 11 chromosomes with an average depth of 64.6 for the wild-type pool and 70.8 for the mutant pool. Of these, 9 located between 31,842,848 nt and 35,015,640 nt were completely or nearly fixed with non-reference alleles (**Figure 3**, **Supplementary Figure 3**, **Table 3**). Of the nine SNPs, two were located in the coding regions of Vigst.04G193900 and Vigst.04G214200 and were predicted to cause single-residue substitutions whereas one was located in the splice donor site of intron 10 of Vigst.04G198600 (**Figure 4A**, **Table 3**). The other six were located in intergenic regions or introns and were not further characterized.

**Table 3:** SNP loci having zero or one wild allele in the mutant bulk of F2 (WT × *isi2*) plants.

Position on chr. 4	SNPs		Depth (wild: mutant)	SNP index at mutant bulk	Description from SnpEff		
	Wild-type	Mutant			Effect	Impact	Gene ID
31,842,848	T	A	0:52	1	Missense	Moderate	Vigst.04G193900
32,352,997	C	T	0:69	1	Splice donor variant	High	Vigst.04G198600
32,970,540	C	T	1:65	0.98	Intergenic	Modifier	–
33,091,871	C	T	0:65	1	Intron	Modifier	Vigst.04G205800
33,941,608	C	T	0:41	1	Missense	Moderate	Vigst.04G214200
34,633,249	C	T	0:57	1	Intergenic	Modifier	–
35,015,358	T	C	0:42	1	Intergenic	Modifier	–
35,015,377	A	G	0:47	1	Intergenic	Modifier	–
35,015,640	A	G	1:35	0.97	Intergenic	Modifier	–

To determine which of the three SNPs is responsible for the *isi2* phenotype, we genotyped 288 F2 (WT × *isi2*) and 163 F3 (WT × *isi2*) plants for the T>A SNP at 31,842,848 nt, the C>T SNP at 32,352,997 nt, and the C>T SNP at 32,970,540 nt on chromosome 4. Complete linkage was found only for the C>T SNP at 32,352,997 nt, whereas recombination was observed between this SNP and both of the other two SNPs (**Table 4**, **Supplementary Table 2**). Data from 163 F3 (WT × *isi2*) plants derived from 11 F2 (WT × *isi2*) plants with recombination among the three SNPs were consistent with the F2 data (**Table 5**, **Supplementary Table 3**). Thus, the C>T SNP at 32,352,997 nt, located in the splice donor site of the 10^th^ intron in Vigst.04G198600, was the single remaining candidate for the *isi2* phenotype.

**Table 4:** Genotypes and phenotypes of recombinants among SNPs in F2 (WT × *isi2*) plants.

Plant ID	SNP at 31,842,848 nt	SNP at 32,352,997 nt	SNP at 32,970,540 nt	Seed water uptake (%)
C035	hetero	mutant	mutant	100
C039	hetero	mutant	mutant	100
C058	wild	wild	hetero	0
C090	wild	wild	hetero	0
C102	hetero	mutant	mutant	100
C107	wild	wild	hetero	0
D036	hetero	hetero	wild	0
D053	hetero	hetero	wild	0
D090	hetero	mutant	mutant	100
D114	hetero	hetero	mutant	0
D142	wild	wild	hetero	0

**Table 5:** Genotypes and phenotypes of recombinants among SNPs in F3 (WT × *isi2*) plants.

SNP at 31,842,848	SNP at 32,352,997	SNP at 32,970,540	Number of individuals	Mean seed water uptake (%)	Mean 100-seed weight (g)	Mean seeds per pod
hetero	Hetero	wild	20	0.50 (2.18)	0.90 (0.11)	10.2 (0.90)
hetero	Hetero	mutant	5	0.00 (0.00)	0.94 (0.07)	8.27 (0.56)
hetero	Mutant	mutant	36	96.0 (6.95)	1.07 (0.11)	4.87 (0.87)
mutant	Mutant	wild	8	92.5 (8.86)	1.01 (0.08)	4.21 (0.86)
mutant	Mutant	mutant	19	93.3 (9.00)	1.11 (0.11)	4.15 (0.97)
wild	Mutant	mutant	15	96.7 (6.17)	1.07 (0.09)	5.14 (1.11)
wild	Wild	mutant	23	0.56 (2.36)	0.95 (0.15)	9.56 (1.75)
wild	Wild	hetero	28	1.92 (4.91)	0.94 (0.14)	9.01 (1.86)
wild	Wild	wild	22	0.00 (0.00)	0.86 (0.08)	10.0 (1.49)

Numbers in parentheses indicate standard deviations.

### 3.4 *VsPSAT1* in the *isi2* mutant and other legumes

**Figure 5:**
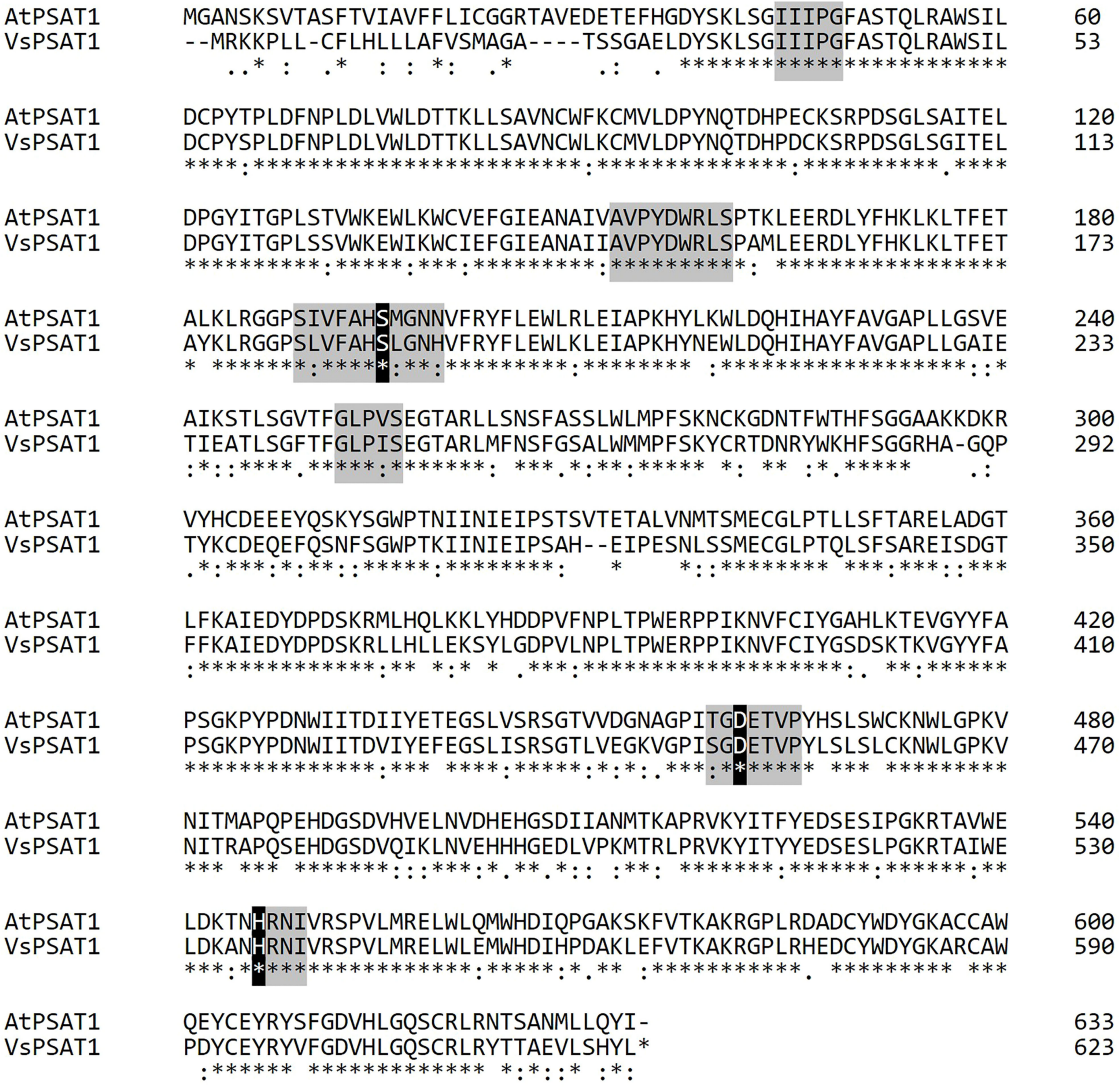
Protein sequence alignment of *V. stipulacea* and *A. thaliana* PSAT1. Asterisks indicate conserved residues. Colons indicate similar residues; periods indicate weakly similar residues. Hyphens indicate gaps introduced to optimize the alignment. Six conserved regions of LCAT-like proteins are highlighted in gray, and the conserved catalytic triad (Ser-Asp-His) is highlighted in black.

A BLAST search identified Vigst.04G198600 as a putative ortholog of the *A. thaliana* gene encoding phospholipid sterol acyltransferase 1 (*AtPSAT1*/*ERP1*, AT1G04010). Therefore, we renamed it *VsPSAT1*. Querying the *V. stipulacea* genome with the amino-acid sequence of AtPSAT1 identified only *VsPSAT1*. The predicted VsPSAT1 protein is 623 residues in length, with an overall identity of 76% with AtPSAT1. The conserved LCAT-like domain (Banas et al., 2005; Lara et al., 2018) has a total of 41 residues in six regions, of which 5 residues in 3 regions were replaced by strongly similar residues, and all 3 residues of the catalytic triad Ser-Asp-His were identical (**Figure 5**).

We performed RT-PCR to determine whether the SNP at the splice donor site affected the mRNA sequence of *VsPSAT1*. The region between the exons 8 and 11 in the *isi2* mutant was approximately 50 bp shorter than that in the wild-type (**Figure 4B**). Sequencing revealed that exon 10 had been skipped (**Supplementary Figure 4**), leading to a frameshift and an ectopic stop codon that truncated the protein from 623 to 452 residues (**Figure 4C**).

We next searched for putative orthologs in species of the tribe Phaseoleae Bronn *ex* DC., specifically other *Vigna* species, *P. vulgaris*, and *G. max*. The diploids (*Vigna* species and *P. vulgaris*) had one copy and the palaeopolyploid soybean had two copies (**Figure 4D**), without any significant degradation in the conservation of the LCAT-like proteins, including the catalytic triad (**Supplementary Figure 5**).

### 3.5 Pleiotropic effects of the *isi2* mutant

**Figure 6:**
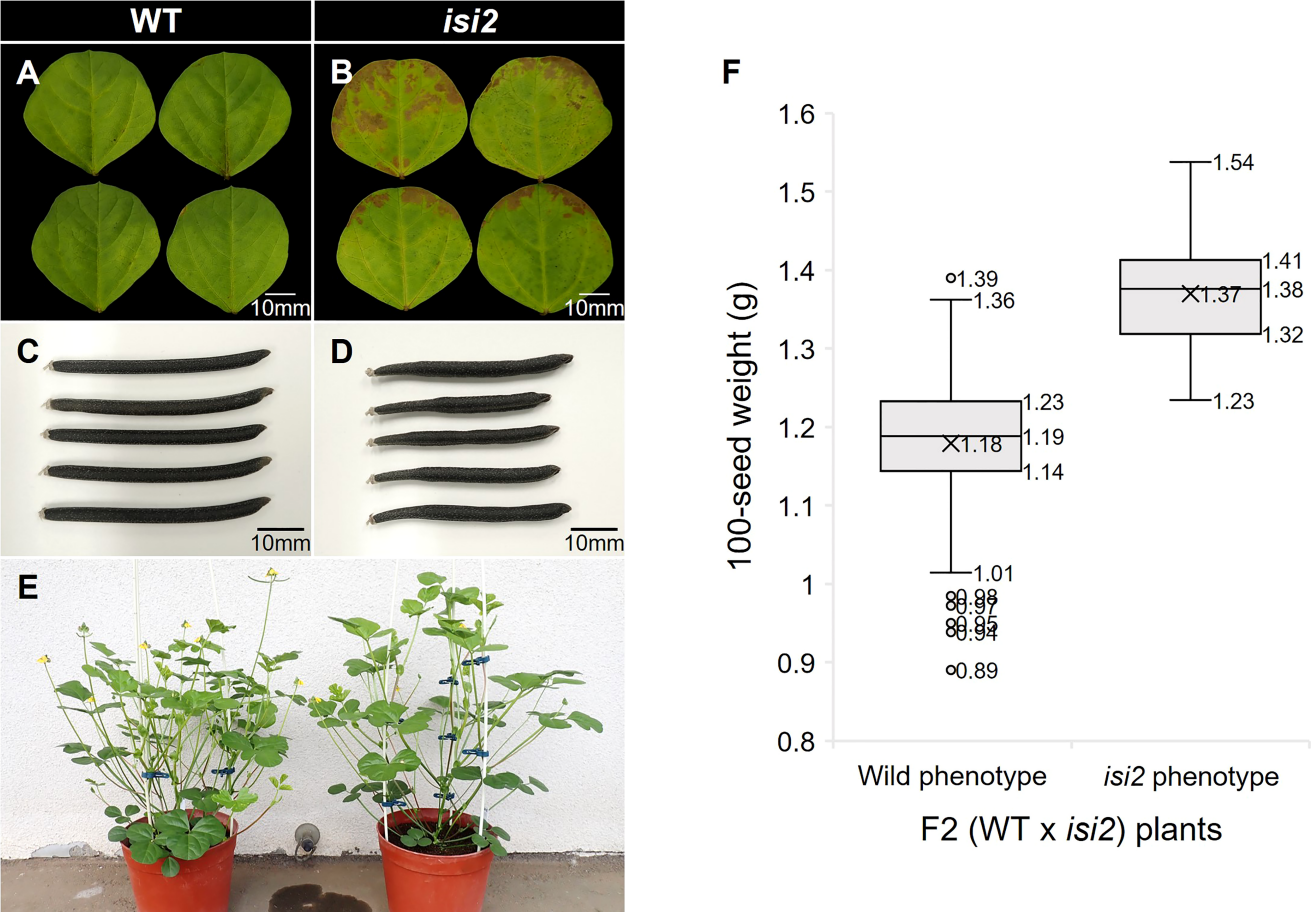
Pleiotropic effects of the *isi2* mutant. **(A, B)** Fully expanded third terminal leaflets were detached from plants and incubated on the water surface for 28 days. N=5. **(C, D)** Mature seed pods of the wild-type and *isi2* mutant, respectively. **(E)** Plants 50 days after sowing. Left, wild-type; right, *isi2* mutant. **(F)** Box-and-whisker plots of 100-seed weights of F2 (WT × *isi2*) plants divided into the wild (N=210) and mutant (N=73) phenotypes. Top, middle, and bottom lines in the boxes indicate the 1st, 2nd, and 3rd quartiles, respectively. Crosses indicate means. Top and bottom lines of the whiskers indicate maximum and minimum values, respectively. Plots indicate outliers.

As the *A. thaliana AtPSAT1* mutant exhibits early leaf senescence due to overaccumulation of sterol esters (Bouvier-Navé et al., 2010), we investigated whether this phenotype occurs in the *isi2* mutant plants. Browning was observed at *isi2* leaflet margins four weeks after detached leaves were floated in water (**Figure 6A, B**). The SPAD value, indicating chlorophyll content, was significantly (*p* < 0.05, *t*-test) different between the wild-type (27.3; SD = 1.37) and *isi2* mutant (20.5; SD = 1.81); however, it was not significantly different between both intact leaves (**Supplementary Table 4**).

We next evaluated the *isi2* mutant for eight traits to elucidate whether it showed pleiotropic effects and found that days to flowering increased by 3 days, the largest leaflet width increased by 7%, the number of seeds per pod decreased by 50%, the pod length decreased by 23%, and the 100-seed weight increased by 37%, compared to the wild-type (**Figure 6C-E**, **Table 6**). No significant differences were observed in height, largest leaf length, and shoot dry weight between the wild-type and *isi2* mutant (*p* > 0.05, *t*-test).

**Table 6:** Wild-type and *isi2* mutant phenotypes for eight agronomic traits (N=7).

Traits	Wild-type		*isi2* mutant		*p*-value from *t*-test
	Mean	SD	Mean	SD	
Days to flowering	43.3	2.3	46.0	0.0	<0.05
Plant height (cm)	21.1	2.7	23.1	1.1	0.09
Largest leaflet width (cm)	7.1	0.3	7.6	0.2	<0.05
Largest leaflet length (cm)	5.9	0.4	6.2	0.2	0.06
Shoot dry weight (g)	6.9	1.1	7.2	0.5	0.62
Number of seeds per pod	12.2	0.4	6.2	1.1	<0.05
Pod length (cm)	4.9	0.1	4.0	0.3	<0.05
100-seed weight (g)	0.94	0.07	1.29	0.06	<0.05

SD, standard deviation.

We next determined if the *isi2 VsPSAT1* mutation was genetically linked with the 100-seed weight and number of seeds per pod. The mean 100-seed weight in 73 *isi2* F2 (WT × *isi2*) plants was 1.38 g, significantly higher than that of 210 wild-type F2 (WT × *isi2*) plants (1.19 g; *p* < 0.05, *t*-test; **Figure 6**, **Supplementary Table 2**). In addition to seed size, the number of seeds per pod was tightly linked to the *isi2* phenotype in 163 F3 (WT × *isi2*) plants derived from 11 F2 (WT × *isi2*) plants with recombination among the 3 SNPs (**Table 5**). However, the linkage of 100-seed weight and number of seeds per pod to the *isi2* phenotype was not complete on an individual basis, owing to high variability (**Supplementary Table 3**).

## 4 Discussion

In this study, we identified *VsPSAT1* as the gene likely mutated to cause the *isi2* phenotype of *V. Stipulacea* plants, increasing water uptake through the lens groove. We hypothesize that *VsPSAT1* functions to close this gap, probably by producing sterol esters. We also identified the pleiotropic effects of the *isi2* mutant: accelerating leaf senescence, increasing seed size, and decreasing numbers of seeds per pod. We assembled the genome of *V. stipulacea* and revealed the differences between the phenotypes of the wild-type and the *isi2* mutant in detail, which we had only approximately evaluated in our previous study (Takahashi et al., 2019). Moreover, we revealed using SEM and CT that the *isi2* mutant has lesser honeycomb-like wax sealing the lens groove than the wild-type, and takes up water from the lens groove. In addition, the *isi2* mutant had pleiotropic effects on other agronomic traits, including organ gigantism in leaves and seeds.

In the present study, we show that *VsPSAT1* is likely the ortholog of *AtPSAT1*/*ERP1* (AT1G04010), whose product catalyzes the transacylation of acyl groups from phospholipids to multiple sterols to produce sterol esters, preventing excessive sterol accumulation. Null *AtPSAT1* mutants have ~30% higher sterol levels in leaves than the wild-type (Shimada et al., 2019). Because sterols are important contributors to cuticular wax in alfalfa (*Medicago sativa* L.) (Lee and Suh, 2015), the loss of *VsPSAT1* function readily explains the *isi2* lens-groove phenotype. However, this phenotype conflicts with that of the *Arabidopsis psat1* mutant, whose seeds exhibit reduced water uptake (Shimada et al., 2021). Abnormal columellar structure in the second layer of the outer integument (oi2) of the seed coat appears to suppress seed water uptake in the *Arabidopsis psat1* mutant (Shimada et al., 2021), and sterols are not detected in the cuticular wax of *A. thaliana* (Lee and Suh, 2015). Moreover, the columella is absent in the seed coat of *V. stipulacea*, and the oi2 layer has honeycomb-like epicuticular wax deposition. Therefore, we hypothesize that differences in seed water uptake between *isi2* and *psat1* mutants are due to differences in seed coat structure and cuticular wax composition, as legumes produce water-impermeable seed coats for physical seed dormancy whereas *A. thaliana* does not. These findings suggest that as *PSAT1* genes are conserved in other wild *Vigna* species, they may act as potential targets for the *de novo* domestication of these species. However, further experiments are required to verify gene function by transformation and chemical analysis.

For *de novo* domestication using reverse genetics or genome editing, it is better to identify multiple genes influencing the same domestication trait, allowing identifications from small, mutagenized populations. Genetic strategies to decrease legume hard-seededness can be divided into two categories: permeabilization of the seed coat and permanent opening of the water gap. In the former case, the genes include *VsCESA7* from the *isi1* mutant in our previous study (Takahashi et al., 2019) and *GmHs1-1* from soybeans (Sun et al., 2015). In the latter case, the genes include *VsPSAT1* from this study and *PAE-8-2* from common beans (Soltani et al., 2021). Of the two categories, counteracting hard-seededness by the permanent opening of the water gap may be more favorable because the *isi2* mutant may provide higher environmental adaptability. In addition, although reduced hard-seededness is necessary for uniform germination, it allows rapid water uptake into the seed immediately after sowing, which causes flooding injury that disrupts soybean seed tissue (Nakayama and Komatsu, 2008), promoting both preharvest germination of pods and fungal infection of seeds in mungbean (*Vigna radiata* (L.) Wilczek), which is closely related to *V. stipulacea* (Humphry et al., 2005). The *isi1* mutant that we previously identified is similar to soybeans, in that it begins to rapidly take up water through the entire seed coat, whereas the *isi2* mutant is similar to common beans in that it exhibits slower water uptake through the lens (Takahashi et al., 2019). In the current global environment with sporadic rainfall and drying cycles, *VsPSAT1* of the *isi2* mutant and *PAE-8-2* of common bean, which contribute to slow water uptake from the lens-specific, are likely more useful genes than *VsCesA7* and soybean *GmHs1-1*. In contrast, *VsCesA7* and *GmHs1-1* are more likely to provide more uniform germination and higher productivity when managed under appropriate environments.

Pleiotropic effects must be considered when selecting target genes for *de novo* domestication. In the present study, the *isi2* mutant showed both agronomically positive traits (decreased hard-seededness and increased seed size) and agronomically negative traits (decreased seed number). Larger seeds are better at lifting the soil and germinating in mungbean and produce plants with a higher shoot and root biomass in soybean (Kluyver et al., 2013; Limede et al., 2018), although the tradeoff between seed size and number is a well-known problem in plant breeding (Dwivedi et al., 2021). The increased seed size and decreased seed number in *isi2* mutants are likely caused by the mutation in *VsPSAT1*, as the SNP and 100-seed weight or the number of seeds per pod were tightly linked in the segregated generations. Moreover, the tight but incomplete linkage likely may have resulted from errors because seed weight is sensitive to environmental factors, and a large number of F2 and F3 plants were cultivated in small pots, which inhibit vegetative growth. Other studies suggest that quantitative or qualitative changes in cuticular wax affect plant growth, including organ size (Zhang et al., 2005; Zhang et al., 2007; Nobusawa et al., 2013; Fukuda et al., 2022; Kajino et al., 2022). In the mungbean, quantitative trait loci (QTLs) for hard-seededness and seed size colocalize (Humphry et al., 2005). We did not discuss seed yield because we did not evaluate the number of pods per plant because complex efforts are needed to estimate seed yield due to its long-lasting flowering and pod-shattering. However, seed yield is one of the most important traits and we will evaluate it in the future. In addition to those traits, the *isi2* mutant also showed early leaf senescence as observed in *AtPSAT1* mutants (Bouvier-Navé et al., 2010). As early leaf senescence shortens the maturation period and prevents green stem disorder in soybeans (Yamatani et al., 2021), we view this phenotype as an agronomically positive trait. Although we detected a significant difference between wild-type and *isi2* mutants for days to flowering, this trait was not linked to an SNP on *VsPSAT1* in F2 and F3 plants (data not shown). To date, few studies have described the pleiotropic effects of domestication-related genes in detail. However, we believe that it is important to catalog both positive and negative pleiotropic effects for better selection of appropriate target genes for *de novo* domestication. Therefore, transformation experiments are needed to overcome the limitations of the present study, such as incomplete linkage.

In addition, our chromosome-level assembly of *V. stipulacea* greatly accelerated our identification of a candidate SNP. The bulked segregant analyses revealed a single clear peak in allele frequency. Moreover, combined with whole-genome sequencing, we identified two candidate genes involved in hard-seededness: *VsCESA7* from our previous study (Takahashi et al., 2019) and *VsPSAT1* in this study. As we previously identified a mutant with non-shattering seed pods (Takahashi et al., 2019), we plan to do another bulked segregant analysis to identify candidate SNPs. We will continue screening for other important traits, including larger seed size and faster growth. As new technologies become available, *de novo* domestication will likely be significantly more rapid and easier than classical breeding to develop climate-adapted crops. We believe that wild legumes, especially those of the genus *Vigna* with pre-existing tolerance to biotic and abiotic stresses, are key to global food security during climate change.

## Data availability statement

The datasets presented in this study can be found in online repositories. The names of the repository/repositories and accession number(s) can be found in the article/supplementary material.

## Author contributions

YT, KN, and NT conceived the study. YT and HS planned the study. CM and YT cultivated plants. HS performed assembly, annotation, BSA, and phylogenetic analysis. HS and KN constructed the genetic map. HE and YT performed SEM. ST and YT performed CT. AH and YT performed RT-PCR. YT performed other experiments. YT, HS, and KN wrote the manuscript with input from TS, ST, AH, HE, and NT. All authors contributed to the article and approved the submitted version.
