# Supplementary material for: Domesticating Vigna stipulacea: Chromosome-Level genome assembly reveals VsPSAT1 as a candidate gene decreasing hard-seededness
Source: Front Plant Sci. 2023 Apr 17;14:1119625. doi: 10.3389/fpls.2023.1119625 (PMC10149957; doi:10.3389/fpls.2023.1119625)
Supplement: Supplementary file 1 [file DataSheet_1.pdf]

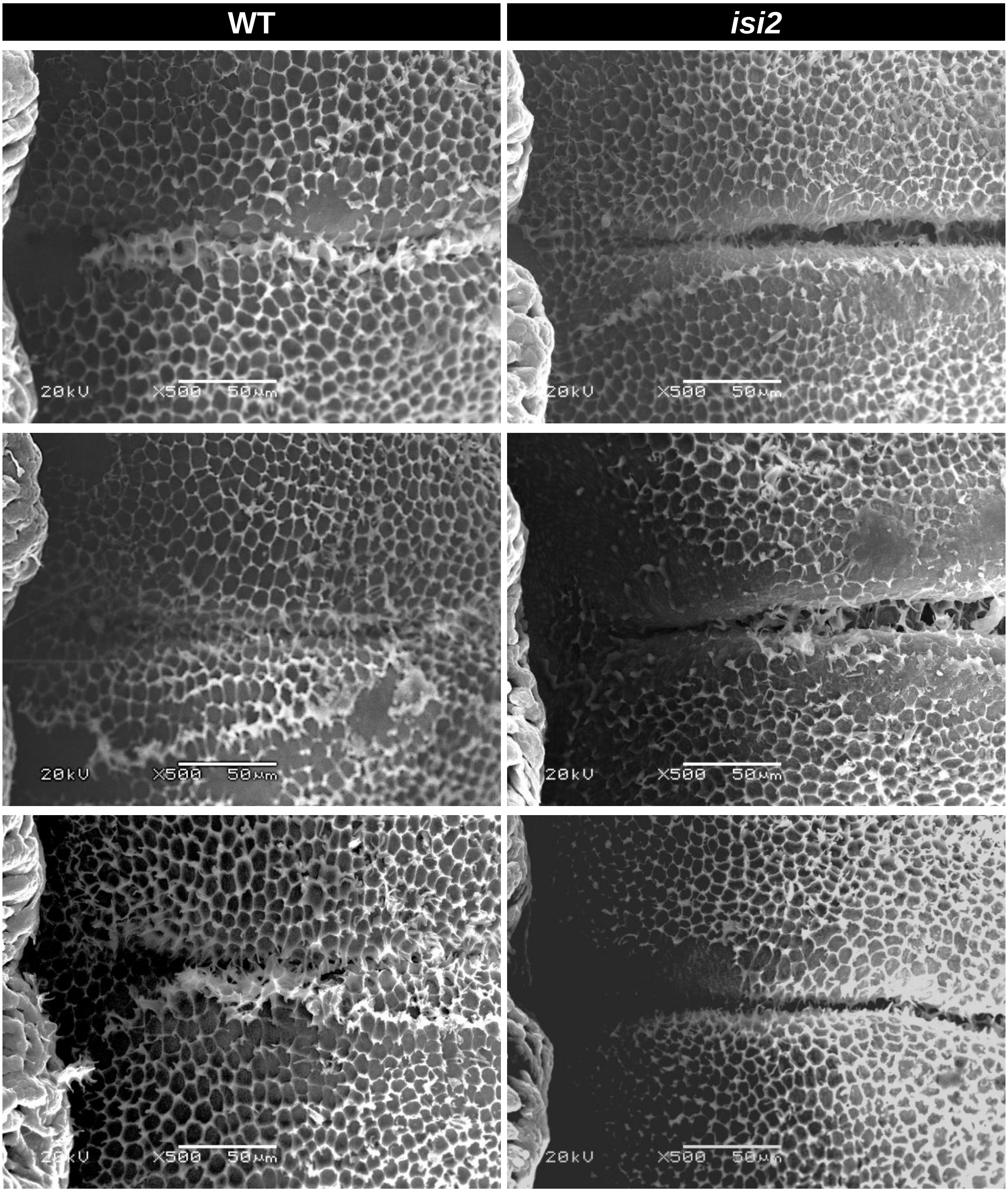

Supplementary Figure 1. 500X scanning electron micrographs around the lens groove of the surfaces of the wild type (left) and *isi2* mutant (right) seeds with three biological replicates each. Bars, 50 µm..

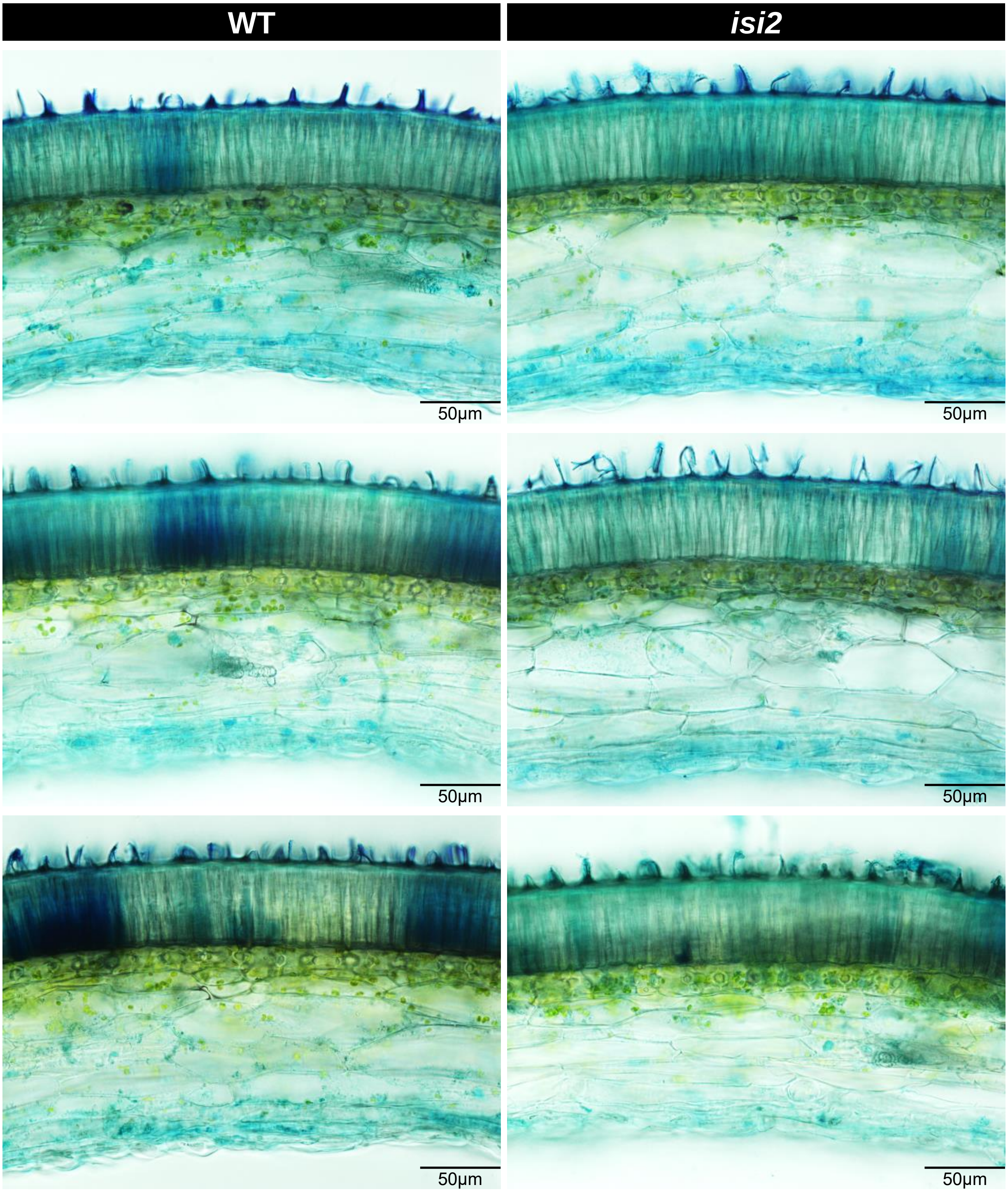

Supplementary Figure 2. Optical micrographs of cross sections of coats of wild-type (left) and the *isi2* mutant (right) seeds with three biological replicates each.

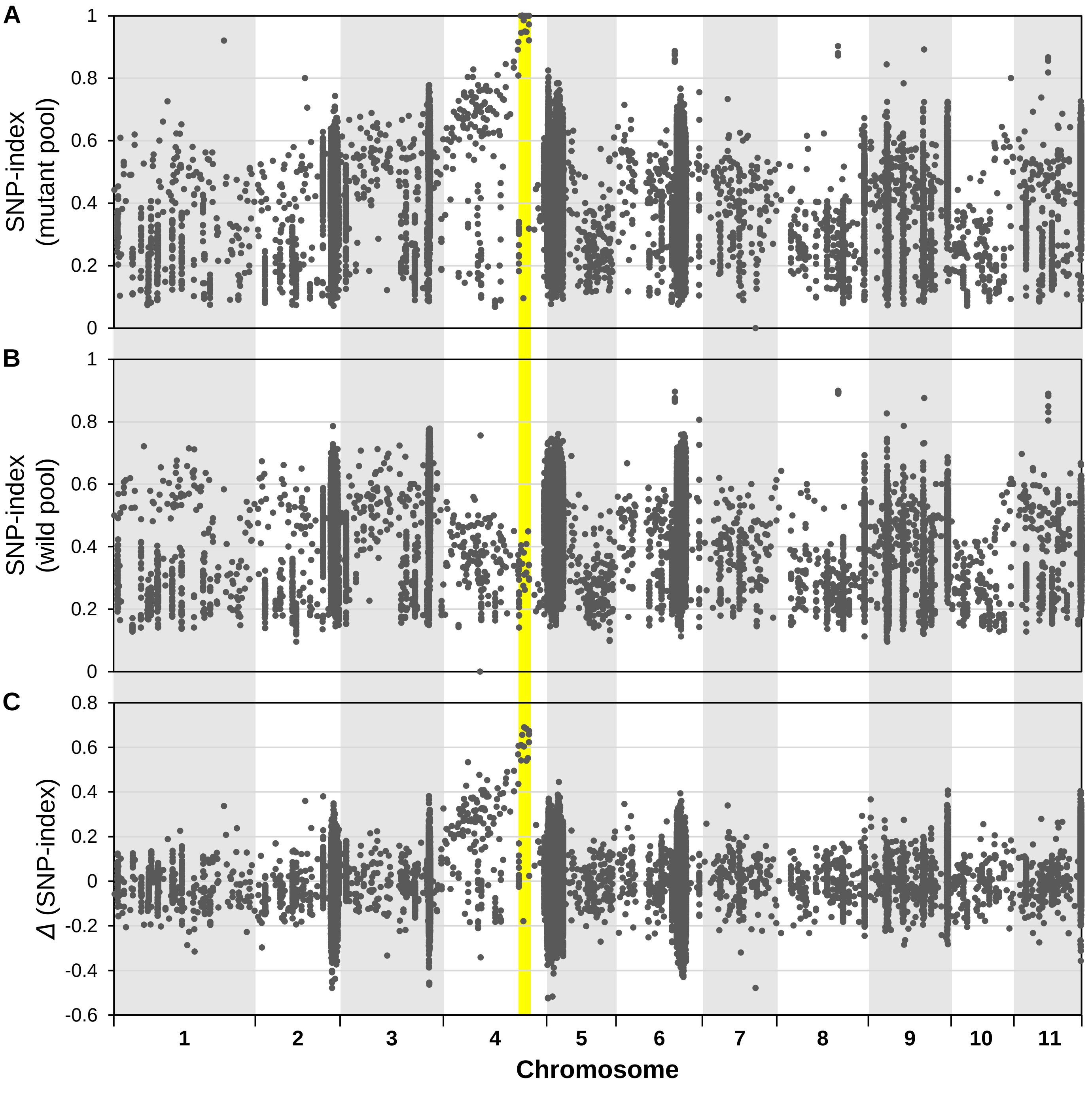

Supplementary Figure 3. SNP-index plot for mutant (A) and wild-type (B) pools of F2 (WT × *isi2*) plants, with the *Δ* (SNP-index) (C). The x-axis indicates chromosomal positions. The y-axis indicates frequencies of the mutant allele (SNP-index) (A, B), and the *Δ* (SNP-index) (C). Yellow highlighting indicates the locus fixed with mutant alleles in the mutant pool.

```
                     9th exon
DNA_WT          AATAGGTTGGTTACTATTTTGCTCCTAGTGGCAAGCCTTACCCCGATAACTGGATCATTACGGATGTCATTTATGAGTTTGAAGGATCTCTAATCTCAAG
DNA_isi2        AATAGGTTGGTTACTATTTTGCTCCTAGTGGCAAGCCTTACCCCGATAACTGGATCATTACGGATGTCATTTATGAGTTTGAAGGATCTCTAATCTCAAG
Transcript_WT   -----GTTGGTTACTATTTTGCTCCTAGTGGCAAGCCTTACCCCGATAACTGGATCATTACGGATGTCATTTATGAGTTTGAAGGATCTCTAATCTCAAG
Transcript_isi2 -----GTTGGTTACTATTTTGCTCCTAGTGGCAAGCCTTACCCCGATAACTGGATCATTACGGATGTCATTTATGAGTTTGAAGGATCTCTAATCTCAAG

DNA_WT          GTACTTCTCTATCTTTGCAGGTGTGTTGGTTTTGTAGTTGTATGTGGAATGAATTGGGGAGAGGGTGGTTTTTGGTTAGAATTTTTGGCACTAACTTTGC
DNA_isi2        GTACTTCTCTATCTTTGCAGGTGTGTTGGTTTTGTAGTTGTATGTGGAATGAATTGGGGAGAGGGTGGTTTTTGGTTAGAATTTTTGGCACTAACTTTGC
Transcript_WT   ----------------------------------------------------------------------------------------------------
Transcript_isi2 ----------------------------------------------------------------------------------------------------

DNA_WT          AAAGTAATATTTTTCTTTTCGTATACAAGGTGTATAATTAGGAAATAGTTATTACTGTTCCAGTTTTTCTGGTTATTCTTTAAAATTAAATGTGTTGAAG
DNA_isi2        AAAGTAATATTTTTCTTTTCGTATACAAGGTGTATAATTAGGAAATAGTTATTACTGTTCCAGTTTTTCTGGTTATTCTTTAAAATTAAATGTGTTGAAG
Transcript_WT   ----------------------------------------------------------------------------------------------------
Transcript_isi2 ----------------------------------------------------------------------------------------------------
                                     10th exon
DNA_WT          TGTTTTAGTCTCAATTGTTAGGTCAGGGACTCTGGTTGAAGGGAAAGTTGGACCTATAAGTGGCGATGAGACGGTAAACTCAAATTATGTTTCTCTCACA
DNA_isi2        TGTTTTAGTCTCAATTGTTAGGTCAGGGACTCTGGTTGAAGGGAAAGTTGGACCTATAAGTGGCGATGAGACGATAAACTCAAATTATGTTTCTCTCACA
Transcript_WT   ---------------------GTCAGGGACTCTGGTTGAAGGGAAAGTTGGACCTATAAGTGGCGATGAGACG---------------------------
Transcript_isi2 ----------------------------------------------------------------------------------------------------

DNA_WT          GTTTTAATTGTTAGGAATGCATAATGTTTTAATTGGCCATTTTTCTACTTCCATCGTTTTTTGAAATTGTGACGTGTGAGAATTACATCTACACTCAACA
DNA_isi2        GTTTTAATTGTTAGGAATGCATAATGTTTTAATTGGCCATTTTTCTACTTCCATCGTTTTTTGAAATTGTGACGTGTGAGAATTACATCTACACTCAACA
Transcript_WT   ----------------------------------------------------------------------------------------------------
Transcript_isi2 ----------------------------------------------------------------------------------------------------
                                              11th exon
DNA_WT          ATTGGATCGCAATAAAATCAACCTTTACAGGTGCCATATCTCTCCCTTTCCTTGTGCAAGAACTGGCTTGGACCAAAAGTGAACATAACAAGAGCGCCTC
DNA_isi2        ATTGGATCGCAATAAAATCAACCTTTACAGGTGCCATATCTCTCCCTTTCCTTGTGCAAGAACTGGCTTGGACCAAAAGTGAACATAACAAGAGCGCCTC
Transcript_WT   ------------------------------GTGCCATATCTCTCCCTTTCCTTGTGCAAGAACTGGCTTGGACCAAAAGTGAACATAACAAGAGCGCCTC
Transcript_isi2 ------------------------------GTGCCATATCTCTCCCTTTCCTTGTGCAAGAACTGGCTTGGACCAAAAGTGAACATAACAAGAGCGCCTC

DNA_WT          AGGTATT
DNA_isi2        AGGTATT
Transcript_WT   AG-----
Transcript_isi2 AG-----
```

Supplementary Figure 4. DNA and cDNA sequences of exons 9, 10, and 11 of *VsPSAT1*. Gray highlighting indicates exon 10. Black highlighting indicates the G>A substitution at the splice donor site between exon 10 and intron 10.

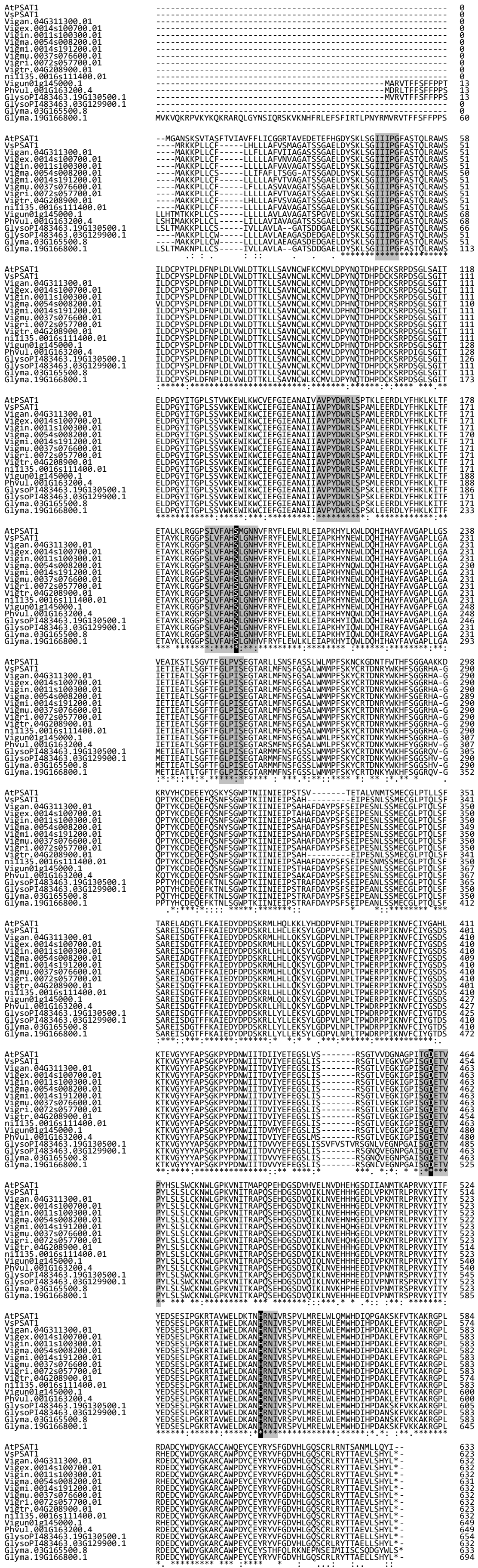

Supplementary Figure 5. PSAT1 protein sequence alignment. Asterisks indicate conserved residues. Colons indicate similar residues; periods indicate weakly similar residues. Hyphens indicate gaps introduced to optimize the alignment. The six conserved regions of LCAT-like proteins are highlighted in gray, and the conserved catalytic triad Ser-Asp-His is highlighted in black.
